# Differentiated Thyroid Cancer in Navarra (Spain): Historic Cohort Results (1987–2003)

**DOI:** 10.5402/2011/560503

**Published:** 2011-07-31

**Authors:** María Pilar Salvador Egea, Ana Aranzazu Echegoyen Silanes, Eduardo Layana Echezuri, Emma Anda Apiñariz, Ana Puras Gil, Edelmiro Menéndez Torre, Lluis Forga Llenas, Amaya Sainz de los Terreros

**Affiliations:** ^1^Department of Surgery, Complejo Hospitalario de Navarra, Irunlarrea street 3, 31008 Pamplona, Spain; ^2^Department of Pathology, Complejo Hospitalario de Navarra, Irunlarrea street 3, 31008 Pamplona, Spain; ^3^Department of Preventive Medicine, Complejo Hospitalario de Navarra, Irunlarrea street 3, 31008 Pamplona, Spain; ^4^Department of Endocrinology, Complejo Hospitalario de Navarra, Irunlarrea street 3, 31008 Pamplona, Spain; ^5^Department of Endocrinology and Nutrition, Hospital Universitario Central de Asturias, Celestino Villamil Street s/n, 33006 Oviedo, Spain

## Abstract

*Introduction*. Navarra has the highest incidence of differentiated thyroid cancer in Spain. The aim of this study was to review its management carried out by the Navarra's multidisciplinary Thyroid Disease Unit, from 1987 to 2003. *Material and Methods*. 325 patients were studied to find the incidence, prevalence, and prognostic factors. Statistical analysis comprised univariate and multivariate Cox proportional hazards regression models for survival and tumor recurrence. *Results*. The average annual incidence was 3.6 per 100,000 inhabitants, with a final prevalence of 82.4 per 100,000. Regarding survival and recurrence, statistical significance was observed for stage IV, follicular carcinoma, capsular and prethyroid muscles invasion, and T4 group. Only survival was related to tumour size larger than 40 mm. Only recurrence was related to lymph node metastases and radioiodine dose higher than 100 mCi. *Conclusions*. Attendance of patients in a functional unit setting has allowed us to classify them into three risk groups.

## 1. Introduction

Differentiated thyroid cancer (DTC) is becoming more frequent. The highest incidence in Spain is found in the region of Navarra [1–3]. Recent research in tyrosine kinase pathogenic activity and inhibitors is becoming more relevant; nowadays, however, related treatments are used only in selected patients [4]. Therefore, currently surgery is the only treatment option that can cure it. If necessary, it can be complemented by radioiodine and suppressive hormone therapy. When very low risk papillary microcarcinoma is detected, it may be sufficient to perform a less extensive surgery [5–8]. On the other hand, when high-risk DTC is found, it is mandatory to complete thyroid intervention with a level VI of Robinson [9] lymphadenectomy because with this approach local recurrence is lower [9–11]. 

Recent changes in guidelines have modified DTC treatment and long-term follow-up. Recombinant human thyrotropin is widely used to avoid levothyroxine treatment withdrawal in thyroglobulin (Tg) detection. High levels of stimulated Tg are considered reliable data and the most sensitive one for early diagnosis of persistent/recurrent disease. A high-resolution ultrasound (US) [12] image obtained by an expert provider can supply important information for diagnosis, staging, and follow-up, and it guides fine needle aspiration cytology (FNAC) [13].

In this study, we set the following objectives.

- To analyse the incidence and prevalence of DTC in the representative population of the region of Navarra.
- To describe demographic characteristics, tumor characteristics, chosen treatment, and results during follow-up (cure, persistent/recurrent disease, and death [14]).
- To evaluate the effect of above-mentioned data on recurrence or mortality, by using statistical analysis.
- To determine prognostic factors; identifying risk groups and, if possible, suggesting criteria focused on the improvement of treatment, management, survival, and low recurrence rate.

Our data have come from the setting of a functional unit, known in our hospital as the “Thyroid Disease Unit.” It is composed of a multidisciplinary team of specialists (surgeons, endocrinologists, pathologists, and radiologists) interested in this field. This Unit treats nearly every patient (more than 90%) suffering from DTC in the public health system of Navarra.

We obtained significant predictive variables in the univariate and multivariate statistical analysis, which are associated with survival and persistent/recurrent disease.

## 2. Methods

Our research is defined as an observational, historic cohorts, descriptive, and analytical study. It includes patients who have been diagnosed with DTC, treated and followed from 1987 until 2003 (17 years) by the Thyroid Disease Unit. From the creation of this unit, data for variables and clinical outcomes have been collected in a database. Following international guidelines, patients were assessed in the out-patient clinic and then the database was updated. The information used was included in the official hospital cancer registry and files, so the process was backed up by local health law and approved by the ethics commitee. Eight patients missed in the follow-up were contacted. They lived in a different region but they were able to provide the required information, therefore they were included in the final results.

In the first five years, our population mean was 518241 people, from 1992 to 1996 it was 520622 people, from 1997 to 2001 it was 567337 people, and the last two years it was 560243 people. In the regional hospital of our area of interest, there was not a public cancer registry available. Nevertheless, we assume that there are few cases detected in it.

Studied variables were as follows.

- Gender and age at the time of surgery. We analysed two groups separately; younger than 45 years old and equal/older than 45 years old at that moment, as indicated in TNM [15–17] staging system.
- Result of presurgical FNAC. We discriminated four groups; suspicious, malignant, not valid, and benign cytology.
- Tumor classification; DTC was classified in papillary carcinoma (PC) or follicular carcinoma (FC) comprising Hürthle carcinoma, as recommended by current pathological classification. Then, we counted PC or FC and their pathological variants if available.
- Tumor size, expressed in milimeters. In seven patients, it was not reported, so those were labelled as Tx.
- Surgical treatment on the thyroid; the kind of surgical procedure of the primary tumor.
- Surgical treatment of cervical lymph node metastases; number of lymphadenectomies performed.
- Surgical complications with in-patients, once they arrived at the surgical ward, were compiled. The most important and frequent ones were recurrent nerve palsy and definitive hypocalcemia after parathyroid injury. Asphyctic hematoma was not included because it usually happens few hours after surgery, and it is managed by anaesthetics.
- TNM staging system at the time of diagnosis. We used the 6th edition pTNM system.
- Thyroid capsule invasion; whether the primary tumor infiltrates into the glandular capsule, meaning an advanced stage, or not.
- Cervical muscle involvement by the primary tumor.
- Unifocal or multifocal tumor in the gland; number of malignant locations in the thyroid.
- Radioiodine treatment; number of treatment sessions and total cumulative dose of I-131 in each patient.
- Positive antithyroglobulin autoantibodies; number of patients affected.

The descriptive study of all these items was executed in a first instance. Later, statistical models were built for analysis of data: univariate model for survival, univariate model for persistent/recurrent disease, multivariate model for survival, and multivariate model for persistent/recurrent disease, always using Proportional Risks Cox Regression. We also show survival curves and the 5- and 10-year survival rates of significant variables. The outcomes of recurrence and persistent disease are grouped together due to their low incidence (below 4%). Also, by doing such statistical analysis it is easier to develop and to understand. The software chosen was SPSS 17.0 for Windows.

## 3. Results

325 patients underwent surgical treatment for their DTC (100%). Annual incidence resulted in 3.6 per 100000 inhabitants, and prevalence was 82.4 per 100000 inhabitants at the time of December 2003.

The ratio for women reached 80%. Our population can be considered young, given that 52.61% were under 45 years old. The presurgical FNAC result was suspicious or malignant in 75%. Regarding tumor classification, PC was diagnosed in 63.38%, with a mean age of 42.90 years old (range 12 to 79 years old) and mean tumor size of 21.75 mm (range 1 to 80 mm). In FC, mean age was 49.56 years old (range 15 to 83 years old) and mean tumor size was 40.89 mm (range 10 to 100 mm). Tumor size was below 40 mm in 79% and below 20 mm in 42%.

Total thyroidectomy with any kind of lymphadenectomy was the surgical procedure in 95.69%, detecting positive lymph nodes in 21.23% of them. Surgical complications collected were as follows: 4% definitive recurrent nerve palsy, 1.8% definitive hypoparathyroidism, and 0.3% surgical wound infection.

Most patients belonged to early tumoral stages; 78.46% were included in stages I and II of TNM system. Details of T and N distribution and stages are shown in Table 1.

Thyroid capsules were affected in nearly 13%, and in 37 subjects, cervical muscle involvement was reported. Multifocal tumors were found in 32.61%. 

Radioiodine treatment was used for 97.53% individuals.

About autoimmunity, we detected positive antithyroglobulin antibodies in 22 patients, with the following characteristics: 90% PC, mean tumor size of 25.9 mm, mean age of 39.5 years old, positive cervical lymph nodes in 59%, and recurrent disease in 9%.

During follow-up, patients were considered to be free of disease when symptoms were absent, physical examination was negative as were image exams, and Tg was undetectable together with thyrotropin higher than 30 mUI/L and negative antibodies. 77.55% of patients fulfilled these criteria. On the other hand, 4% of patients have died (8 with PC and 5 with FP) and 3.69% presented with recurrent disease.

Finally, the final 14.76% corresponded to subjects that had some biochemical abnormality during follow-up, usually a single elevation of thyroglobulin, but could not be included in the cured group nor in those who had tumor evidence on radiology exams. Distant metastases were detected in 9 (2.8%) patients. The most frequent target was lung; 8 patients (89% of metastatic disease). In four of them, lung metastases coexisted with bone (3 patients) or brain (1 patient) location. One patient diagnosed of multiple metastatic sites suffered from subcutaneous spread. Every distant spread was diagnosed after first surgical treatment. The summary of descriptive data is shown in Table 1.

In the univariate model for survival, we found statistical significance in the variables; capsule invasion, prethyroid muscle involvement, tumor size larger than 40 mm, T4 group of TNM (with regard to the T1 group and to the “rest of T” group: T1+T2+T3), and stage IV (with regard to the “rest of stages”). They predicted a higher risk of mortality. Results are shown in Table 2.

In the univariate model for recurrent disease, we also found statistical significance in the variables; capsule invasion, prethyroid muscle involvement, positive cervical lymph nodes, T4 group of TNM (with regard to the T1 group and to the “rest of T” group; T1+T2+T3), and stage IV (with regard to the stage I and to the “rest of stages”), and total cumulative dose of radioiodine higher than 100 mCi. Results are shown in Table 3.

**Figure 1:**
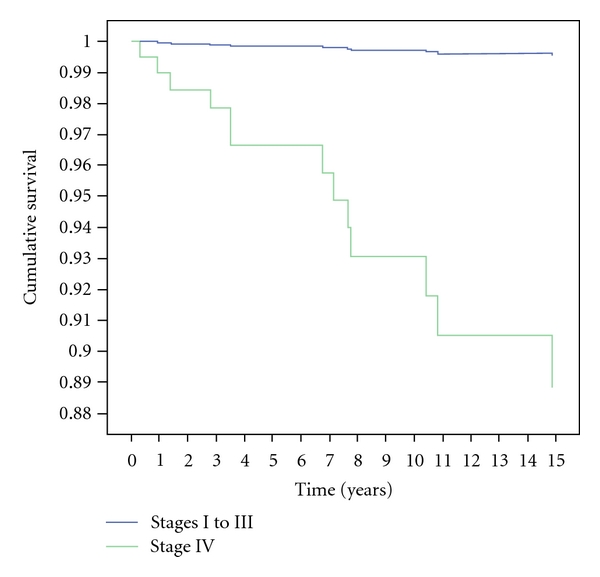
Survival curve of stage IV and rest of stages in the multivariate analysis.

**Figure 2:**
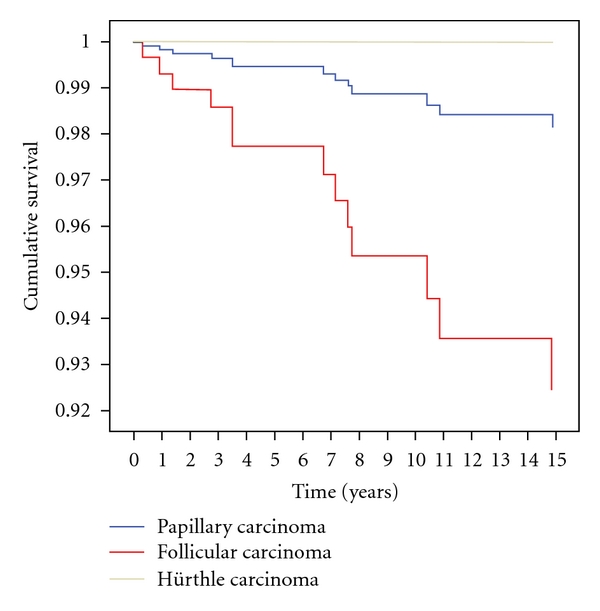
Survival curve of pathological classification in the multivariate analysis.

In the multivariate model for survival, higher mortality was related to stage IV compared to the “rest of stages” and related to FC compared to PC. Results are shown in Table 4. Survival curves are represented in Figures 1 and 2. 

In the multivariate model for recurrent disease, higher risk of recurrence was observed in patients with prethyroid muscle involvement, positive cervical lymph nodes, and FC compared to PC. Results are shown in Table 5. 

The 5-year survival rate and the 10-year survival rate of each significant variable are given in Table 6.

## 4. Discussion

In Spain, the highest incidence of DTC has been described in the region of Navarra. Values adjusted for age are 3.10 and 9.36 per 100000 inhabitants/year in men and women respectively, between 1987 and 1997. In Germany, France, and Italy [1], similar results were obtained due to early diagnosis and thorough analysis of surgical tissues. 

Our research included patients diagnosed, treated, and followed for 17 years in a Thyroid Disease Unit. Therefore, we got quite a good number of subjects and the management by a functional unit made possible to use identical protocols and to initiate little changes according to current external guidelines. Also, patients dying because of their DTC may have died during those 17 years and recurrences or persistent disease should have been evident, because two thirds of those situations appear within the 10 years following surgery [16, 17]. That long-term follow-up is a positive aspect of our study to bear in mind [14]. 

In relation to gender, we were able to prove that women were more affected (80%), with a women/men ratio of 4/1, somewhat higher than previously described; Ponce Marco [2] and Sitges Serra and Sancho Inserser [3] have reported a ratio of 2/1, without providing any explanation for differences. Gender distribution in the general population cannot justify it because in Navarra and in their region it is similar. Even though female life expectancy is longer than male's in our area, DTC [18–23] was detected mainly in young people. 

Most of the people were young. Scheiden et al. [24] have observed similar data in Luxemburg (1990–1999); 310 subjects had a mean age of 48.3 years old, with a PC increase from 1997 due to an early diagnosis in opinion of the authors. Our association between age and survival was nearly statistically significant. Given that more than 50% were younger than 45 years old and none of them had died during follow-up, we obtained a distorted statistical model. So, we propose that young age could have an important positive predictive value on survival. 

Regarding diagnosis, FNAC is considered the test of choice by expert panels [2, 3]. In our work, it has been malignant or suspicious in 75%, with an increased value when performed in recent years probably because high-resolution US [25] was introduced for early detection of local recurrence, for diagnosis of lymph node metastases, and for guiding FNAC [26]. At the end of the study, we obtained a false positive result in 1% and a false negative result in 5%. 

In our patients, most tumors were PC. However, we found a more elevated rate of FC than in previous studies [2, 3, 18–23, 27]. Its cause remains unknown to us. Perhaps it could be explained by a higher prevalence of FC in immigrants or stronger environmental factors for goiter in some areas of our region. We advise supplementing diet with iodine and updating epidemiological research focused on understanding it.

We did not find statistical association between surgical procedure and survival. Except for 14 patients, in every case total thyroidectomy was executed, with subsequent radioiodine treatment if needed and hormone suppression [28].

The number of lymphadenectomies registered is not too high (66 cases). Nevertheless, we must remember that routine cervical level VI dissection in papillary carcinoma was widely accepted not long before this study was finished. For Mazzaferri and Kloos [29], mediastinal and cervical bilateral lymph node metastases modify recurrences and survival as independent predictors and specific surgery on lymph areas improves those items. In our series, cervical lymph node metastases impair recurrences but not survival. This tumor stage has traditionally been treated surgically, both in the initial diagnosis and lymph node recurrence. 

Surgical morbidity keeps on being a worrying topic. Sancho Fornos et al. [23] have published a meta-analysis about benign and malignant goiter surgery; in best series, he found 1.5% nerve injury and 0–2% definitive hypocalcemia. But when level VI lymphadenectomy is associated, nerve injury rises to 3–4% and hypocalcemia reaches 14–17%. In our work, there is an intermediate morbidity in relation to those previous results. We believe that factors that might contribute are the observational nature of the study (historic cohort), several surgeons and resident surgeons implicated in treatment, and the later recommendation of level VI lymph node dissection [30].

Looking back to the relevance of age within stages and early diagnosis for prognosis, it is worthwhile remembering that more than half of the population were under 45 years old. Moreover, diagnosis came earlier in most cases (63.69% belong to stage I). These circumstances may explain the survival for both kinds of DTC. Our survival rate can be related to previous works; Beasley et al. [31] have reported no deaths in stage I and a mean age in patients dying from their DTC of 68.5 years old.

Interpretation of negative effect of a radioiodine treatment dose over 100 mCi on persistent disease can be understood because more aggressive tumors force us to use higher doses of it. Also recurrences are treated with several sessions of radioiodine and in that way the total dose is increased. Even though this variable showed statistical significance as mentioned before, we do not include it in Table 3 because of the chronological opposite relationship. Analysis of positive antithyroglobulin antibodies did not reach statistical significance, maybe because of the low sample size. However, those tumors seemed to be more aggressive after studying descriptive data. 

Studying outcomes according to former characteristics, every subject developing persistent/recurrent disease was diagnosed with PC (12 cases). Ten of them were under 45 years old. Within related aggressive factors, we can describe 6 patients with worse PC variants (2 of tall-cells and 4 of diffuse sclerosant), 7 with positive cervical lymph adenopathies at diagnosis, 5 with thyroid capsule infiltration, 5 were multifocal tumors, and 5 with prethyroid muscle involvement. In all of them, persistent/recurrent disease consisted of locoregional spread (thyroid bed and cervical lymphadenopathies), and so were surgically treated. Stojadinovic et al. [32] have reported the follow-up of 431 recurrent DTC during 13 years, finding a 35% local recurrence, 23% local and regional recurrence, and 30% local and distance recurrence. Symptoms were evident only in 26%. They conclude that survival can be predicted by age under 45 years old, subclinical or local recurrence, and the ability of maintaining the disease-free situation. Mazzaferri and Jhiang [14] have published a follow-up of 30 years and show a survival of 76% and 30% recurrences. Mortality increased every decade over 40 years old. The recurrent disease appeared more often in age under 20 and over 59 years old. In our series, DTC was the cause of death in every patient suffering from distant metastases. We counted 13 (4%) DTC total deaths; 9 (2.8%) with distant disease and 5 (1.5%) with local recurrence. Only one patient died suffering from local and distant recurrence. When patients showed symptoms or were classified in stage IV at the time of diagnosis, we observed the above-mentioned bad prognosis. This group had a mean age of 68.3 years old. 

Some workshops and expert panels have suggested risk classifications, both through a few parameters for grouping patients and through many clinical data providing complex systems [33, 34]. Probably, the most used risk classification for clinicians during follow-up is the one based on basal or stimulated Tg detection. Mazzaferri et al. [33] have defined a group of disease-free patients when Tg is below 0.5 ng/mL (60–70% of patients), a group of patients needing close follow-up if Tg is 0.6 to 2 ng/mL (15–20%), and a group likely to show local or distant recurrence or persistent disease if Tg is over 2 ng/mL (20–25%). Schlumberger et al. [34] have also supplied risk factors for recurrence; tumor size larger than 20 mm and/or with cervical locoregional spread. Prospective studies have shown that patients with undetectable Tg and negative cervical US show a less 0.5% risk of 10-year recurrence rate. Sánchez Franco [13] has demonstrated that this risk increases as tumoral stage does. This statement can be considered a brief general concept, present in many studies.

## 5. Conclusions

Our results allow us to classify patients in three risk groups defined as follows.

- A group with a better long-term prognosis would be composed of subjects younger than 45 years old, with PC diagnosed in FNAC, and with DTC detected in early stages (T1 to T3, smaller than 40 mm, negative cervical lymph nodes and without capsule or muscle involvement).
- A group with a higher risk of recurrent disease, but not higher mortality, would be represented by those patients with positive cervical lymph nodes and those who have received a total cumulative dose of radioiodine more elevated.
- A group with higher risk of recurrence and death would be composed of patients of 45 years old or older, with FC and advanced stage (stage IV, T4 or larger than 40 mm, with capsule or muscle invasion).

In our opinion, this classification could help in guiding treatment and follow-up; frequency of out-patient visits and need of complementary examinations, being aware of methodological limits from any observational research.

##  Conflict of Interests

None of the authors presents any conflict of interests.

## Figures and Tables

**Table 1:** General overview of main descriptive results.

Variables and their classifications	Ratio
Age: younger than 45 years old	52.61%
Gender: women	80.00%
Presurgical FNAC: suspicious or malignant	75.00%
Tumor pathological classification:	
(i) Papillary carcinoma (mean age 42.90 years old, mean tumor size 21.75 mm)	63.38%
(ii) Follicular carcinoma (mean age 49.56 years old, mean tumor size 40.89 mm)	36.61%
Surgical treatment on the thyroid: total thyroidectomy	95.69%
Surgical treatment of cervical lymph node metastases	21.23%
Surgical complications:	
(i) Recurrent nerve palsy	4.00%
(ii) Definitive hypoparathyroidism	1.80%
TNM classification:	
(i) T1: 125 patients	38.46%
(ii) T2: 97 patients	29.84%
(iii) T3: 61 patients	18.76%
(iv) T4: 42 patients	12.92%
(v) N0: 256 patients	78.76%
(vi) N1: 69 patients	21.23%
Stages:	
(i) I: 207 patients	63.69%
(ii) II: 48 patients	14.76%
(iii) III: 40 patients	12.30%
(iv) IV: 30 patients	9.23%
(a) IV a: 21 patients	6.40%
(b) IV c: 9 patients	2.80%
(v) Stage I and II	78.46%
Radioiodine treatment	97.53%
Positive antithyroglobulin autoantibodies	6.77%
Complete remission	77.55%
Mortality	4.00%
Persistent/recurrent disease	3.69%

FNAC: fine needle aspiration cytology.

**Table 2:** Univariate model for survival.

Univariate model for survival	*P*	Hazard ratio	HR 95% CI	
			Lower	Upper
Age	0.056	79.9	0.8	7157.7
Thyroid capsule invasion	0.000	9.2	3.1	27.5
Muscle involvement	0.000	12.1	4.0	36.4
Tumor size; >40 mm versus <20 mm	0.033	10.3	1.2	88.3
T4 versus T1 (TNM)	0.003	24.1	2.9	195.9
T4 versus rest of T (TNM)	0.000	10.9	3.4	34.5
Stage IV versus rest of stages	0.000	20.3	6.6	62.3

**Table 3:** Univariate model for persistent/recurrent disease.

Univariate model for persistent/recurrent disease	*P*	Hazard ratio	HR 95% CI	
			Lower	Upper
Thyroid capsule invasion	0.000	5.3	2.7	10.2
Muscle involvement	0.000	6.9	3.6	13.4
Positive lymph nodes	0.000	3.7	1.9	7.0
T4 versus T1 (TNM)	0.000	4.3	2.0	9.4
T4 versus rest of T (TNM)	0.000	5.4	2.8	10.5
Stage IV versus rest of stages	0.001	3.5	1.6	7.4

**Table 4:** Multivariate model for survival.

Multivariate model for survival.	*B*	*P*	Hazard ratio	HR 95% CI	
				Lower	Upper
Stage IV versus rest of stages	3.284	0.000	26.674	8.643	82.322
Follicular versus papillary ca.	1.439	0.012	4.216	1.366	13.008
Hürthle versus papillary ca.	−13.556	0.983	0.000	0.000	

**Table 5:** Multivariate model for persistent/recurrent disease.

Multivariate model for persistent/recurrent disease	*B*	*P*	Hazard ratio	HR 95% CI	
				Lower	Upper
Muscle involvement	1.851	0.000	6.366	3.106	13.047
Positive lymph nodes	1.154	0.002	3.172	1.520	6.619
Follicular versus papillary ca.	0.821	0.049	2.274	1.005	5.146

**Table 6:** Survival rates of significant variables.

Variable	5-year survival rate (%)	10-year survival rate (%)
Stage IV	96.7	93
Rest of stages	99.9	99.8
Follicular carcinoma	97.7	95.3
Papillary carcinoma	99.5	98.8
Thyroid capsule invasion	92	85
Thyroid capsule not invaded	99	98
Muscle involvement	90	82
Muscle not involved	99	98
Tumor size >40 mm	95.5	94
Tumor size <20 mm	99.6	99.3
T4 (TNM)	91.5	86
Rest of T (TNM)	99.5	99
T1 (TNM)	99.6	99.3

## References

[1] Carvajal VL, Santamaría MP (2005). Epidemiología del cáncer diferenciado de tiroides. *Endocrinologia y Nutricion*.

[2] Ponce Marco JL, Garcia JLL (2005). Cáncer de tiroides. *Cirugía AEC: Manual de la Asociación Española de Cirujanos*.

[3] Sitges Serra A, Sancho Inserser J, Serra AS, Inserser JS (1999). Carcinoma diferenciado de tiroides, carcinoma medular de tiroides, carcinoma anaplásico de tiroides y linfoma tiroideo. *Guías Clínicas de la Asociación Española de Cirujanos*.

[4] Riesco-Eizaguirre G, Santisteban P (2007). Molecular biology of thyroid cancer initiation. *Clinical and Translational Oncology*.

[5] Schlumberger M, Berg G, Cohen O (2004). Follow-up of low-risk patients with differentiated thyroid carcinoma: a European perspective. *European Journal of Endocrinology*.

[6] Torre EM, Carballo MTL, Erdozáin RMR, Llenas LF, Iriarte MJG, Layana JJB (2004). Prognostic value of thyroglobulin serum levels and ^131^I whole-body scan after initial treatment of low-risk differentiated thyroid cancer. *Thyroid*.

[7] Biondi B, Filetti S, Schlumberger M (2005). Thyroid-hormone therapy and thyroid cancer. *Nature clinical practice. Endocrinology & Metabolism*.

[8] Sherman SI, Angelos P, Ball DW (2005). Thyroid carcinoma. *Journal of the National Comprehensive Cancer Network*.

[9] Jiménez AL, Serra AS (2005). Cáncer diferenciado de tiroides. Avance en el tratamiento quirúrgico. *Endocrinología y Nutrición*.

[10] Machens A, Dralle H (2010). Decreasing tumor size of thyroid cancer in Germany: institutional experience 1995–2009. *European Journal of Endocrinology*.

[11] Machens A, Dralle H (2010). Failure to consider the number of dissected sides can bias complication rate calculations of central lymph node dissection for thyroid cancer. *Archives of Surgery*.

[12] Frasoldati A, Pesenti M, Gallo M, Caroggio A, Salvo D, Valcavi R (2003). Diagnosis of neck recurrences in patients with differentiated thyroid carcinoma. *Cancer*.

[13] Sánchez Franco F (2005). Directrices en el tratamiento del carcinoma diferenciado de tiroides. *Endocrinologia y Nutricion*.

[14] Mazzaferri EL, Jhiang SM (1994). Long-term impact of initial surgical and medical therapy of papillary and follicular thyroid cancer. *American Journal of Medicine*.

[15] Rosai J, Carcangiu ML, Ronald A, Delellis MD, Rosai J, Sobón LH (1992). *Atlas of Tumor Pathology: Tumors of Parathyroid Glands*.

[16] Pacini F, Schlumberger M, Dralle H, Elisei R, Smit JWA, Wiersinga W (2006). European consensus for the management of patients with differentiated thyroid carcinoma of the follicular epithelium. *European Journal of Endocrinology*.

[17] Cooper D, Doherthy G, Haugen B (2006). Manegement guidelines for patients with thyroid nodules and differentiated thyroid cancer. American Thyroid Asociation. *Thyroid*.

[18] Witt RL, McNamara AM (2002). Prognostic factors in mortality and morbidity in patients with differentiated thyroid cancer. *Ear, Nose and Throat Journal*.

[19] Zambudio AR, Rodríguez JM, Moya R (2002). Los alelos HLA-CW7 y CW1 como factores de mal pronóstico en el carcinoma diferenciado de tiroides en el sudeste español. *Cirugia Espanola*.

[20] Kinder BK (2003). Well differentiated thyroid cancer. *Current Opinion in Oncology*.

[21] Mazzaferri EL (2000). Long-term outcome of patients with differentiated thyroid carcinoma: effect of therapy. *Endocrine Practice*.

[22] Mazzaferri EL (1999). An overview of the management of papillary and follicular thyroid carcinoma. *Thyroid*.

[23] Sancho Fornos S, Urbaneja JV, Marco JLP, Jiménez RP, Vela CH (2001). Complicaciones de la cirugía tiroidea. *Cirugia Espanola*.

[24] Scheiden R, Keipes M, Bock C (2006). Thyroid cancer in Luxembourg: a national population-based data report (1983–1999). *BioMed Central Cancer*.

[25] Sheth S, Hamper UM (2008). Role of sonography after total thyroidectomy for thyroid cancer. *Ultrasound Quarterly*.

[26] Rago T, Vitti P (2008). Role of thyroid ultrasound in the diagnostic evaluation of thyroid nodules. *Clinical Endocrinology and Metabolism*.

[27] Sherman SI (2003). Thyroid carcinoma. *The Lancet*.

[28] Shaha AR (2004). Implications of prognostic factors and risk groups in the management of differentiated thyroid cancer. *Laryngoscope*.

[29] Mazzaferri EL, Kloos RT (2001). Current approaches to primary therapy for papillary and follicular thyroid cancer. *Journal of Clinical Endocrinology and Metabolism*.

[30] Ready AR, Barnes AD (1994). Complicaciones de la tiroidectomía. *British Journal of Surgery*.

[31] Beasley NJP, Walfish PG, Witterick I, Freeman JL (2001). Cause of death in patients with well-differentiated thyroid carcinoma. *Laryngoscope*.

[32] Stojadinovic A, Shoup M, Nissan A (2002). Recurrent differentiated thyroid carcinoma: biological implications of age, method of detection, and site and extent of recurrence. *Annals of Surgical Oncology*.

[33] Mazzaferri EL, Robbins RJ, Spencer CA (2003). A consensus report of the role of serum thyroglobulin as a monitoring method for low-risk patients with papillary thyroid carcinoma. *Journal of Clinical Endocrinology and Metabolism*.

[34] Schlumberger M, Pacini F, Wiersinga WM (2004). Follow-up and management of differentiated thyroid carcinoma: a European perspective in clinical practice. *European Journal of Endocrinology*.

